# An Exploratory Analysis of Collective Patterns of Conscious Experience Using a Self-Report Questionnaire

**DOI:** 10.3389/fpsyg.2021.634677

**Published:** 2021-08-13

**Authors:** Ovidiu Brazdau, Sona Ahuja, Cristian-Dan Opariuc, Valita Jones, Sadhna Sharma, Carlo Monsanto, Sperry Andrews, Keith Fiveson

**Affiliations:** ^1^Consciousness Quotient Institute, New York, NY, United States; ^2^Faculty of Education, Dayalbagh Educational Institute, Agra, India; ^3^Faculty of Law and Administrative Sciences, Ovidius University, Constanta, Romania; ^4^Office of Success Coaching (OSC), California State University San Marcos, San Marcos, CA, United States; ^5^Kendriya Vidyalaya Sangathan, New Delhi, India; ^6^Iolee Ecosystem, South Richmond Hill, NY, United States; ^7^Human Connection Institute, Santa Fe, NM, United States; ^8^Work Mindfulness Institute, New York, NY, United States

**Keywords:** conscious experience, patterns, self-report, consciousness quotient, conceptual convergence, consciousness, meta-research, inventory

## Abstract

This study is an exploration of collective patterns of conscious experience, as described by various psychological models, using a self-report questionnaire: The Consciousness Quotient Inventory (CQ-i). The CQ-i evaluates patterns of behaviors, attitudes, and attentional styles as well as the usage of conscious skills, awareness, and the capacity to “feel awake and alive,” providing a complex exploration of conscious experience. A set of 237 items covering major aspects of the subjective conscious experience was selected to detect the phenomenal patterns of subjective conscious experience. An exploratory factor analysis on a large sample (*N* = 2,360), combined with our previous meta-research on conceptual convergence of conscious experiences, revealed that these experiences appear to have 15 patterns common to all of us. A sample with a quasi-normal distribution (*n* = 2,266) was employed for standardization and classification of scores (*M* = 100; *SD* = 15). The study provides a conceptual framework for future in-depth studies on collective patterns of self-awareness, inner growth dynamics, and psychological maturity.

## Introduction

The concepts of consciousness and states of consciousness have been debated across different scientific communities including psychology and cognitive science, philosophy, neuroscience, psychiatry, and physics (Tart, 1975; Baars, 1986, 1999; Wolman and Ullman, 1986; Damasio, 1989; Natsoulas, 1992; Penrose, 1994; Crick and Koch, 1995; Chalmers, 1996; Cohen and Schooler, 1997; Hameroff et al., 1998; Varela and Shear, 1999; Metzinger, 2002). Throughout the 20th century, western psychology has understood self-consciousness as an adaptive personality process that entails the natural human disposition of becoming an object of ones’ own consciousness (DaSilveira et al., 2015).

In the consciousness studies literature, there are significant arguments showing that the concept of “consciousness” needs refinements. Some authors consider that consciousness is a “mongrel” concept, cobbled together of distinct phenomena with little more than the label “consciousness” in common (Block, 2007) and, to advance, consciousness science would need to split our current notion into more finely crafted properties (Klein and Hohwy, 2015).

There is an increased tendency to use philosophy and neuroscience to explain states of consciousness and leave deep psychology aside. Bernard Baars felt the need to emphasize this issue decades ago, by writing a paper titled *There is already a field of systematic phenomenology, and it’s called Psychology*, where he mentions that “the things we humans can report accurately are the same things we experience as conscious! Reportability is the generally accepted index of consciousness. In point of fact, therefore, psychologists are always asking people about their conscious experiences” (Baars, 1999, p. 217). From a cognitive-behavioral perspective, conscious processes have been operationally defined as events that can be reported and acted upon with verifiable accuracy and under optimal reporting conditions (Baars et al., 2003).

Still, the terms “conscious” and “unconscious” are quite difficult to define and use, and the researchers studying cognition and neural correlates have recently stopped using them and opted for “explicit/implicit” (Kehr, 2004; Thrash et al., 2012; Runge and Lang, 2019), while the methods for capturing the neural correlates of conscious experience have reached a very high level of sophistication. Amid this excitement about the neural correlates, there has been comparatively little systematic attention paid to the behavioral methods used in scientific studies of consciousness. A focus on behavioral, psychophysical methods is considered to be “a welcome corrective” (Klein and Hohwy, 2015). Some researchers even consider that “consciousness is hard to study” is a kind of self-fulfilling prophecy, because consciousness scientists focused so much on the hardness of the problem, sometimes at the cost of the development of empirically adequate theories, giving rise to this illusion of inherent scientific inadequacy (Lau and Michel, 2019).

The *comprehensive measurement* of conscious experience with assessment instruments has been a rare topic in psychological research, perhaps because psychologists could not find a method to reach a satisfactory level of significance of the results (Natsoulas, 1992; Petitmengin, 2009). Yet, there are many assessment instruments that have been in use for some time, with good results in evaluating various *facets* of the conscious experience; for example, Self-Consciousness Scale (Fenigstein et al., 1975), Psychological Well-Being Scales (Ryff and Keyes, 1995), The Phenomenology of Consciousness Inventory (Pekala, 1982), Mindfulness Attention and Awareness Scale-MAAS (Brown and Ryan, 2003), Freiburg Mindfulness Inventory (Walach et al., 2001, Toronto Mindfulness Scale (Bishop et al., 2004), Kentucky Inventory of Mindfulness Scale (Baer et al., 2004), Revised NEO Personality Inventory (Costa and McCrae, 1992), Emotional Quotient Inventory (Bar-On, 1997), Self-Compassion Scale (Neff, 2003), Multidimensional Assessment of Interoceptive Awareness (Mehling et al., 2012), Mystical Experience Questionnaire (MacLean et al., 2012), Leadership Maturity Assessment Instrument and Loevinger’s Washington University Sentence Completion Test (Cook-Greuter, 2000), Troyer Level of Consciousness Inventory (Troyer, 2012), and various transpersonal psychology scales (MacDonald and Friedman, 2002).

The usability of some of these measures is still to be confirmed. In a study on self-consciousness concept and assessment in self-report measures (DaSilveira et al., 2015), five self-report questionnaires were analyzed: Self-Consciousness Scale Revised (Scheier and Carver, 1985), Self-Reflection and Insight Scale (Grant, 2001), Self-Absorption Scale (McKenzie and Hoyle, 2008), Rumination-Reflection Questionnaire (Trapnell and Campbell, 1999), and Philadelphia Mindfulness Scale (Cardaciotto et al., 2008). The results were not as expected, as Private Self-Consciousness, Public Self-Consciousness, Private Self-Absorption, and Public Self-Absorption did not react as expected in most analyses. On the other hand, the maladaptive/adaptive distinction involving the Rumination, Private Self-Absorption, and Public Self-Absorption scales was confirmed (DaSilveira et al., 2015).

The researchers studying altered states of consciousness (ASC) have been very interested in using self-report questionnaires for measuring features of the conscious experience. A systematic literature research of psychometric tools which assess the subjective experience during ASCs (Schmidt and Berkemeyer, 2018) revealed a multitude of scales from various tests that can be used to explore phenomenological patterns of conscious experience, such as oceanic boundlessness, dread of ego dissolution, visual restructuralization, spiritual experience, impaired control and cognition, elementary imagery, experience of unity, audio-visual synaesthesia, disembodiment, changed meaning of percepts, anxiety, complex imagery, insightfulness, somaesthesia, ineffability, positive mood, and transcendence of time and space.

The gold-standard in quantitative experimental research to measure ASC experiences is the retrospect assessment with standardized and validated questionnaires (Schmidt and Berkemeyer, 2018). Multiple questionnaires have been developed to quantify different aspects of ASC phenomena. The researchers studying ASC concluded that an ASC is not a mere quantitative change in a single cognitive function (e.g., elevated arousal), instead, it is a multidimensional phenomenon, meaning that not only one aspect of consciousness is affected, but the relative intensity of multiple consciousness aspects changes. In their review paper on ASC assessment tools, Schmidt and Berkemeyer conclude that such “phenomenological patterns” can be operationalized as the factor structure of the applied psychometric assessment, i.e., the individual ratings, or factor scores, of a questionnaire. Such psychometric measures allow direct comparisons between induction methods, individual’s responses, averaged group responses, and different experimental settings (Schmidt and Berkemeyer, 2018).

Due to the inherent limitations of the self-report questionnaires to explore subjective experiences, introducing the Consciousness Quotient (CQ) construct as a comprehensive descriptor of conscious experience was an extended and delicate task.

To better detect and understand the patterns of conscious experiences, a meta-research aiming at conceptual convergence of conscious experiences from an experiential perspective was undertaken between 2012 and 2018 (Brazdau, 2018). The underlying assumption was that it is necessary to cover significant areas of conscious experience and to include the development and transformation of conscious skills and capacities as a result of the inner growth process and psychological maturity. Conceptual convergence has been previously used successfully in various areas of research, such as digital literacy, learning, and communication (Weinberger et al., 2007; Toma, 2014; Martínez-Bravo et al., 2020). We similarly used the method, seeking to understand the contribution of each term to an integrated conceptualization of conscious experience, while using the “conscious experience” as a multidimensional process that connects and integrates all the facets and processes we observed during meta-analysis. The knowledge was then used to generate the questionnaire content.

During this meta-research (Brazdau, 2018), a variety of sources were analyzed: literature research (consciousness studies, articles, and books); mindfulness research; psychometrics research; spiritual wisdom; personal experience of non-dual people, interviews on Conscious TV, and the “Buddha at the gas pump” websites; personal experience of witnessing awareness; experts, friends, psychologists, and research partners; discussions on the “Consciousness Science” Google group discussions (a group composed of more than 60 consciousness experts, mostly speakers at the “Toward a Science of Consciousness” conferences), and Google suggestions (e.g., we analyzed the first 100 websites that referred to “I am aware of” and checked the recommended links as a source of collective knowledge, especially first-person data). Some concepts used in consciousness studies were explored and included as descriptors/traits of conscious experience: mindfulness (Baer et al., 2006), post-autonomous ego development (Cook-Greuter, 2013), witnessing awareness (Brazdau, 2014), meta-awareness/awareness of awareness itself (Monsanto, 2013), and emotional intelligence (Heim, 2003).

Related concepts and studies were analyzed and considered to find adequate descriptors of conscious experience, including attention regulation (Fehmi, 2003; Monsanto, 2013), neuroplasticity (Hanson, 2011), metacognition (Darling-Hammond et al., 2014), rational/irrational belief-dynamics (Vasile, 2012), spiritual intelligence (King, 2008), conceptual systems and personality organization (Harvey et al., 1961), affective neurosciences (Davidson and Begley, 2012), “enlightenment” and “awakening” experiences (Costeines, 2009), persistent non-symbolic experiences (Martin, 2010), neurotheology (Murphy, 2002), levels of human development (Graves, 1970), outrospection (Krznaric, 2014), human connectedness, unity consciousness and embodied love (Andrews, 1996; Andrews and Salka, 2014; Andrews and Tayloe, 2015; Beichler, 2016), Ubuntu philosophy (Gianan, 2010), non-dual awareness (Josipovic and Malach, 2006), pure awareness (Forman, 1990; Genarro, 2008), psychedelics research (Kent, 2010; Fadiman, 2011), spiritual crises (Grof, 2009), orchestrated objective reduction theory (Hameroff, 2010), critical reviews of consciousness studies (Blackmore, 2010), and advaita and neo-advaita philosophy (Conway, 2008).

Along with conscious experience, two additional concepts were fundamental to our research: awareness and attention. We expanded on the interpretation provided by Brown and Ryan (2003, p. 822): awareness is “the background radar of consciousness, continually monitoring the inner and outer environment. One may be aware of stimuli without them being at the center of attention. Attention is a process of focusing conscious awareness, providing heightened sensitivity to a limited range of experience. In actuality, awareness and attention are intertwined, such that attention continually pulls figures out of the ground of awareness, holding them focally for varying lengths of time.” Our analysis of the collective patterns of attention also include explorations of the attention schemes/styles used by individuals (Graziano, 2013) that provide valuable insights into the functioning of the narrow-focused attention and wide-receptive attention. Attention to attention, a rare attentional style (Fehmi, 2003) was also analyzed.

The researches in developmental psychology were significantly beneficial for our study, especially the theories and researches on mature ego development (Graves, 1970; Loevinger, 1976; Kegan, 1994; Cook-Greuter, 2000; Martin, 2010; O’Fallon, 2015; Wilber, 2016), which allowed us to understand better the dynamics of conscious experience patterns as they transform from one stage to another. The distinction between states (temporary identity configurations) and stages (habituated structures) was also relevant, as it provided a conceptual framework for understanding the transformation of the conscious experience over time. For instance, the attention to attention (meta-attention) or awareness of ego as a system may be accessed as temporary experiences in the conventional ego development stages, but they habituate in the post-conventional stages.

The results of the conceptual meta-research regarding the psychological facets of conscious experience and its transformation patterns are included in the Psychology of Becoming Conscious e-book (Brazdau, 2018), providing the necessary conceptual modeling for the CQ construct. The conceptual research contains four parts: inner growth journeys, awakening journeys, patterns of transformation, and psychological explanations of inner growth challenges that are currently labeled as psychiatric conditions.

Owing to this in-depth meta-research on conceptual convergence of conscious experiences, the CQ construct was developed using a sample of more than 300 traits, skills, and abilities that were later translated into 287 items and situations. To establish the content validity of the CQ-i index of conscious experiences, the list was analyzed by a group of experts (Brazdau, 2015; Brazdau and Ahuja, 2016). The goal was to evaluate the comprehensiveness and representativeness of the content, whether the lists of traits that compose the inventory adequately cover conscious experience, with no irrelevant content included (Newton, 2013). Of the 108 invited people, the final panel comprised 25 members with relevant personal and professional experience related to inner transformation and becoming more conscious through various transformative practices. The study confirmed that the CQ-i had appropriate content that adequately covered conscious experience. Forty items were excluded from the CQ-i, and 27 items were re-worded to better suit the traits they measured. Some panelists raised concerns about its length and ease of use, which were monitored over time. According to the suggestions we received from panelists, we added 45 items to cover some areas that were not explicitly explored by the CQ-i items.

Consequently, new terminological clarifications were developed to describe the CQ construct: “To be conscious is to have a degree of witnessing awareness and a degree of freedom of choice when thinking, feeling, sensing, and interacting with people and the environment. ‘More conscious’ (a higher CQ) means a higher degree of witnessing awareness and being less automatic in thinking-feeling-sensing, together with a higher degree of choice when initiating a behavior.” “Witnessing awareness” is usually described as the “I am experience,” “the observer experience,” “just being” (as opposed to “doing”), “aware of awareness itself,” or “no-mind.” “Mindfulness” is a related construct (right mindfulness—*samma sati* in Buddhism—seems connected to witnessing awareness); however, in modern mindfulness, as promoted in the West, being mindful does not go beyond being a cognitive observer. To clarify this distinction, the term “non-conceptual self” was proposed (Brazdau, 2014), as a part of personal identity that has witnessing awareness as its primary function, complementary to the “conceptual self,” which has cognition as its main function.

To further test the behavior of the items, the new questionnaire was released online, and the assessment platform was adapted to the new length. In this stage, we collected feedback from participants regarding the final version of the items. We analyzed a feedback dataset with 358 entries, referring mainly to the wording of the items and, in some cases reporting the inappropriateness of the item for their lives. As a result, minor wording adjustments were made to improve the brevity of some items, while the items perceived as unclear or inappropriate were completely removed from the questionnaire, resulting in the final version of the questionnaire, comprising 237 items that were used in the data collection for this study.

In 2016, as the online dataset grew (*N* = 1,568), an evaluation was performed to determine how many patterns/factors the new CQ-i could detect. A preliminary Exploratory Factor Analysis (EFA) showed a model with many small dimensions (>40 dimensions with an eigenvalue > 1), and a larger sample was necessary to interpret the results correctly; thus, we continued the online data gathering. Preliminary data also showed that conscious experience modeling must have highly inter-related factors instead of independent factors, as we previously hypothesized (Brazdau, 2009).

Between 2016 and 2019, a series of exploratory studies using the paper-pencil version of the CQ-i questionnaire were undertaken by researchers from the Dayalbagh Educational Institute and other organizations in India, providing more data for understanding the measurement (Das, 2016; Kumar and Singh, 2016; Pyari and Srivastava, 2016; Sahni and Kumar, 2016; Misra et al., 2017; Kumar and Pawar, 2018; Upadhyay et al., 2018; Nigam and Misra, 2019; Saxena et al., 2019).

## Materials and Methods

### Research Objectives

The development of the CQ-i questionnaire was a step-by-step process, between 2003 and 2020, following these main objectives: item development (e.g., clarity of the content, format of the items, relevance to conscious experience, validity and reliability, and discriminative power); response scale development (Likert scales and yes/no formats were tested); usage of reversed items; developing lie filters and other options to check for fake answers; developing a conceptual-experiential factor model, focused on the functionality of the factors and relevance for the inner growth process; and compliance with international assessment standards.

During the CQ-i development, the test had various forms and lengths:

• CQ-i beta version (preliminary set of 62 items): six factors in the CQ model.
• CQ-i v. 2014 (287 items): 257 items in Section 1, measured with a Likert scale; 30 items in Section 2 with yes/no answers; and eight factors in the CQ model.
• CQ-i v. 2015 (273 items): 243 items in Section 1 and 30 items in Section 2.
• CQ-i v. 2016 (249 items): 237 items in Section 1 and 12 items in Section 2.
• CQ-i v. 2020 (268 items): 237 items in Section 1 and 31 items in Section 2. Both sections use a six-level frequency Likert scale.

A timeline of the CQ-i development steps and the list of the previously published papers are provided in Table 1.

**TABLE 1:** Timeline of the CQ-i development studies.

**Timeline**	**Population/scientific activities**	**Results/decisions**
2003–2007	Literature research, item pool, evaluating other scales	Selection of 64 items
2007	*N* = 150, paper-pencil	Two items removed and 15 items reformulated; CQ-i beta version = 62 items
2008	*N* = 2,474 50% paper–pencil, 50% online	Publication of Ovidiu Brazdau’s Ph.D. thesis, socio-demographics correlations, and body types correlations; CQ-i Beta Version available online (until 2012)
2009–2010	Consultation with experts and conference discussions	Feedback from experts: Harris Friedman, Bernard Baars, Jonathan Shear, and John Rowan 2009 Toward a Science of Consciousness conference presentation, Hong Kong
2009–2011	*N* = 5,464, online, 5-point Likert scale *N* = 112, online, 6-point Likert scale	Sample composition: Romanian = 5,012, English = 1,168
	*N* = 65, paper–pencil	Changed from a 5-point to a 6-point Likert scale (kept agree-disagree format) to adjust for the observed tendency of participants to mark the answer in the middle of the scale when unsure about the answer
May 2011	*N* = 70, paper–pencil; military population	Discussions
2011	*N* = 30, paper–pencil	Discussions
2011	*N* = 120, paper–pencil, general ability measure for adults	Conference paper: Brazdau and Mihai, 2011. The Consciousness Quotient: A new predictor of students’ academic performance. Procedia – Social and Behavioral Sciences, 11, 245–250
2011	*N* = 145, paper-pencil	2011 Toward a Science of Consciousness conference presentation, Stockholm
January–August 2012	Library research. Five researchers in the research team: Petru Constantinescu, Ramona Sbircea, Iuliana Constantinescu, Andreea Butucescu, and Sofia Dumitriu	Re-checked all the items using 24 criteria Each conscious behavior searched for in the scientific literature Compliance with APA, ITC, and ETS standards Evaluation for introducing validity scales: Lie Scale, Acquiescence Scale, and Extremity Response
September 2012	*N* = 12, Willis’ Cognitive Interviewing; Likert scale evaluation: revised from agreement to frequencies	PsiWorld 2012 Conference paper published in Procedia – Social and Behavioral Sciences: Consciousness Quotient Inventory improvement: Qualitative study using cognitive interviewing approach (Brazdau et al., 2013) All items were revised so that the statements would refer to behaviors instead of attitudes The Likert scale was modified to a six-level frequency scale
October 2012	*N* = 120, paper–pencil	Updated eight items and life situations/conscious behaviors
December 2012	*N* = 102, paper–pencil	All reversed items were removed (*n* = 12) Statistical analysis; new items for the Lie Scale
February 2013–August 2014	In-depth construct development and content validity research	The content validity was evaluated by a panel of 25 experts. Consequently, 40 items were excluded, and 27 items were re-worded New terminological clarifications were developed to operationalize the CQ construct better. Re-introduced reversed items, experimentally (13 items) CQ-i v. 2014 beta version released (287 items: 257 items in Section 1, 30 items in Section 2) “The Consciousness Quotient: Construct development and content validity research”—paper presented at PsiWorld 2014 conference (published in Procedia – Social and Behavioral Sciences, 187, 244–249) CQ-i Administration on adolescents: Difficulty level assessment”—conference paper published in Procedia – Social and Behavioral Sciences, 128, 387–392
April 2015–October 2016	*N* = 632 Items analysis; extended validity and reliability research	Adjusting items based on feedback from online testing (*N* = 358) Statistical analysis, 14 items removed; testing new Lie Scale, four reversed items CQ-i v. 2015 released (273 items; 243 items in Section 1, 30 items in Section 2)
June–August 2016	*N* = 1,312	No more reversed items Six items removed from Section 1, 18 items removed from Section 2 Some small modifications to words in some items, for better comprehension; lie scale—four pairs; CQ-i v. 2016 released (249 items; 237 items in Section 1, 12 items in Section 2) Preliminary factor analysis showing the axis structure with many small dimensions; waiting for a larger sample
March 2017	*N* = 1,568	Statistical and psychometric analysis Data follows the same trend with many small factors; decision to continue gathering data.
2018–2020	*N* = 2,360 Normative sample = 2,266	Exploratory factor analysis Reliability analysis CQ-i v. 2020 released, 268 items (237 items in Section 1, 31 items in Section 2).

*CQ-i, Consciousness Quotient Inventory; APA, American Psychological Association; ITC, International Test Commission; ETS, Educational Testing Service.*

Considering all the previous steps, the research objectives for this study were as follows:

• to detect and describe the collective patterns of conscious experience, using EFA and the previous conceptual research;
• to adjust the theoretical model of conscious experience to the collective patterns detected by EFA;
• to evaluate if the collective patterns can be used as scales/factors, and to create a modular assessment structure for future researches;
• to design a standardized reporting system, useful for people who use the CQ-i assessment for self-discovery, by providing a friendly and meaningful scoring classification.

### Sample and Procedure

In October 2019, the dataset collected online since 2014 (*n* = 2,471) was exported from the online assessment platform at www.consciousness-quotient.com. During data collection, no financial gratification was offered to participants for filling the questionnaire, and the order of questions was fixed for each participant. All the participants completed the CQ-i assessment in the English language. Sample items are available in Appendix.

After discarding possible invalid answers, a dataset of 2,360 entries was used in the EFA procedure. After the EFA procedure and following a decision to tighten the inconsistency filters (using five pairs of two semantically consistent items in each pair), 94 entries were further removed; thus, a dataset of 2,266 entries was used for norming and score classification. All data analyses were performed with SPSS (IBM, Armonk, NY, United States) and Jamovi (Open Source Project^1^).

The primary socio-demographic data are described below (*n* = 2,266; *M* = 36.35 years; *SD* = 12.49 years; age range = 82 years, ages 18–100 years old):

• Age intervals (years): 18–19, 5%; 20–29, 29.1%; 30–39, 30.5%; 40–49, 20.2%; 50–59, 10.2%; 60–69, 3.7%; ≥70, 1.3%;
• Sex: women, 63.7%; men, 36.3%;
• Countries: 96 (Romania, 46.5%; United States, 18.3%; India, 5.6%; Great Britain, 4.9%; Australia, 2.8%; Canada, 2.3%; Mexico, 2.3%; South Africa, 1.8%; and 15.6% from 88 other countries);
• Highest education: college/university, 43.9%; master’s degree, 31%; high school, 15.4%; Ph.D./doctoral degree, 5.8%; grade school, 1.3%; not applicable, 2.6%.

To calculate the global score and the scales score, the sum of all item ratings was used. The scoring scale was ranged as, Almost never (definitely no): 1 point; Very rarely/Once in a while: 2 points; Occasionally/Seldom: 3 points; Quite often/Sometimes: 4 points; Very frequently/Usually: 5 points; Almost always (definitely yes): 6 points.

## Results

### Exploratory Factor Analysis (*N* = 2,360)

**FIGURE 1:**
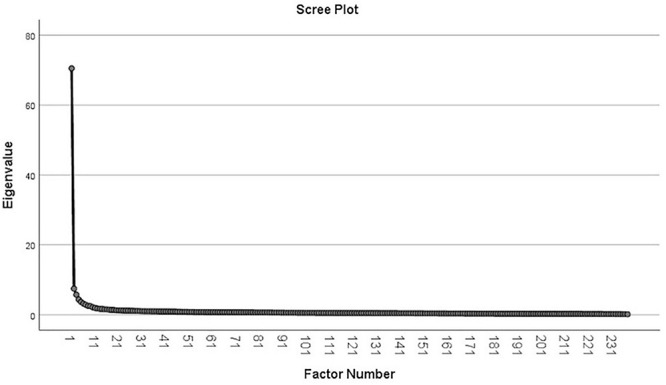
Scree plot (*N* = 2,360).

The principal axis analysis (with Oblimin rotation for correlated factors), using the Kaiser criterion (eigenvalue > 1) revealed 37 main dimensions, with a cumulative eigenvalue of 58.983. The data are presented in Table 2 (Axes loadings) and Figure 1 (Scree plot).

**TABLE 2:** Principal axis analysis (*N* = 2,360).

**Axis**	**Keywords**	**Total**	**Initial eigenvalues**	
			**% of variance**	**Cumulative %**
1	Self-identity awareness, various patterns	70.521	29.756	29.756
2	* Openness and self-love (perhaps an impulsive pattern)	7.477	3.155	32.911
3	Emotional, energy body changes, psychosomatic awareness	5.747	2.425	35.336
4	Feel, post-conventional, spiritual-related, witnessing, interconnectedness	4.384	1.850	37.186
5	Social-relational, people interactions awareness, flexibility	3.670	1.549	38.734
6	Awareness of smell and food	3.204	1.352	40.086
7	* Feeling people’s energy and flowing with life	2.873	1.212	41.298
8	Aware of language use and social interactions using an autonomous or post-autonomous style	2.561	1.080	42.379
9	Self-reflection in social interactions	2.476	1.045	43.423
10	Social discussions, overview awareness, present-moment awareness, energy, interconnectedness, comfort	2.131	0.899	44.323
11	Post-autonomous, mindfulness, clarity	1.963	0.828	45.151
12	Deep interpersonal connections, easy connect, mindfulness, post-autonomous style, aware of experience details while interacting	1.811	0.764	45.915
13	Awareness of inner growth, analyze life moments, events, self-systems, recognize	1.685	0.711	46.626
14	Post-autonomous style, aware of language limits, and subjectivity of experience	1.655	0.698	47.324
15	Self-identity awareness, related to “intuition”	1.577	0.666	47.990
16	Feel/aware of others’ energy/dynamics	1.541	0.650	48.640
17	Post-autonomous style construct aware, mindfulness, large perspective, highly self-aware, thoughts, emotions	1.468	0.619	49.259
18	(Multi)perspective flexibility, allowing contradictions/opposites, post-autonomous style construct aware, cognitive-attentional openness	1.410	0.595	49.854
19	Witnessing, self-observing, notice subpersonalities, mindfulness, a degree of inner peace, time for self-reflection (inner growth practice active, perhaps)	1.383	0.583	50.437
20	An overall relational-social-based post-autonomous style, spiritual, flexibility, perspective, meta-view, noticing emotions, patterns	1.296	0.547	50.984
21	Physical awareness, detect and notice, sounds, smells, colors, energy plus aware of attention, witnessing (construct aware and unitive)	1.263	0.533	51.517
22	Negative, body-physical awareness (smells, taste, color etc.) related to identity); opposite to mindfulness—perhaps a pulsional pattern	1.246	0.526	52.043
23	* Authenticity, observing, quick learning, and present-moment awareness	1.221	0.515	52.558
24	Nature, ecological awareness, spiritual interconnectedness, physical, post-autonomous unitive style	1.199	0.506	53.064
25	Social-relational and identity analytical skills, construct aware, aware of language, perspective, mindfulness	1.185	0.500	53.564
26	Identity, post-autonomous style unitive, feel energy dynamics awareness, spiritual, mindfulness present-moment, awareness, human beings—a global perspective, wonder, full of meaning	1.150	0.485	54.050
27	Notice automatic patterns of thinking-speech, some feel-body related (breathing), identity, aware of attention styles	1.145	0.483	54.533
28	Flexibility of emotional identity, non-attachments, self-compassion, resilience, no-drama, unitive, witnessing, mindfulness	1.122	0.473	55.006
29	Non-conceptual feel, just being, energy openness, acceptance, oneness, unitive and mindfulness, present-moment awareness, human beings	1.110	0.468	55.474
30	Mindful living, autonomous style, authentic, comfortable with discomfort and neutral experiences, emotional acceptance, identity energy, and information	1.093	0.461	55.935
31	Fresh perspective, feel moments, kindness, wonderful, wonder, post-autonomous, openness to new experiences, non-conceptual awareness, good awakeness (aware in dreams)	1.069	0.451	56.386
32	Depth of interconnectedness, connection with people and life, accept new things, energy and social awareness, flexible empathy with people	1.046	0.441	56.828
33	Growth awareness, practice, self-analysis, life changes, change habits, detecting patterns (90% information-related patterns)	1.036	0.437	57.265
34	Big picture, information, overview, notice, social-relational perspective awareness, and construct aware style	1.029	0.434	57.699
35	Just three items related to mindfulness and detecting nuances	1.019	0.430	58.129
36	Detecting social-relational cues, emotion-based, social intelligence, and aware of its dynamics	1.017	0.429	58.558
37	* Aware of social-relational dynamics, cognitive, detecting social deception, and social catalyst; perhaps an automatic social pattern	1.007	0.425	58.983

*Extraction method: Principal axis factoring, Oblimin rotation. The terms autonomous, post-autonomous, construct aware, and unitive refer to ego maturity stages (Cook-Greuter, 2013). Items marked with “^∗^” have negative correlations, probably reflecting collective impulsive/automatic patterns.*

The patterns were analyzed and identified, then aggregated into composite scales, per their loading. According to the parallel analysis using the 95 percentile criterion (*N* = 2,360), the number of components to retain was 16, including all values that were larger than the corresponding random eigenvalues (Horn, 1965; Hayton et al., 2004; Patil et al., 2017). The axes analysis showed that a model with 14 scales would be optimal (not 16 as recommended by the parallel analysis), as all the 37 axes fit into 14 patterns, and there was no need for additional fragmentation.

The mapping procedure was developed mainly considering the loadings > 0.5. For loadings between 0.3 and 0.5, the allocation was performed using the meta-research on conceptual convergence of conscious experiences (Brazdau, 2018). All observable patterns detected in the axes provided by EFA were analyzed, as they were already observed during the meta-research; for instance, perspective-taking was included in the conceptual research as a mechanism for configuring the conscious experience (Brazdau, 2018). As the patterns are inter-related; most of the items were allocated to more than one scale. Mapping data are presented in Table 3 (Mapping axes/patterns).

**TABLE 3:** Mapping axes to patterns/scales (*N* = 2,360).

**Patterns of conscious experience**	**No. of items**	**Axes detected by the exploratory factor analysis**
Perspective-taking	61	9, 17-a, 18-a, 20-a, and 34
Clarity of discrimination	107	11 and 37
Quality of experience	41	30-a and 31
Spirituality-harmony	36	4-a, 20-b, and 24-b
Global self-identity	101	1, 2, 4-b, 17-b, 23, 26, 30-b, and 35
Language use	42	8, 14, and 17-c
Physical self	36	6, 21, 22, and 24-a
Energy self	38	7 and 28
Cognition self	65	5-a, 15 and 25-a
Non-conceptual self	31	19-a and 29
Social-relational interconnectivity	86	5-b, 10, 12, 16, 20-c, 25-b, 32, and 36
Inner growth	81	13, 18-b, 19-b, and 33
Multi-modal integration	43	3
Habitual patterns	21	27
Awakening skills	30	*Selection based on research (experimental scale)

*Axes marked with -a, -b, -c, -d are included in more than one scale.*

A new scale, Awakening Skills, was introduced experimentally to test unusual configurations of conscious experience. Thirty existing items were allocated to this scale based on conceptual research and EFA data, and a new section with 31 items was added to the questionnaire, to explore exclusively this category of rare experiences.

### Patterns Description

The following descriptions are intended to illustrate the complexities of each pattern of conscious experience. Future conceptual studies are necessary to provide clearer descriptions and definitions for some of the scales.

#### Perspective-Taking (61 Items)

Perspective-taking is the process by which a person filters reality using various vantage points, or various lenses through which they select information sources and create meaning. This is a complex process, involving attention, cognition, perception, and other systems of conscious experience (Monsanto, 2013). Usually, the perspectives are culturally conditioned (Cook-Greuter, 2000).

#### Clarity of Discrimination (107 Items)

Clarity of discrimination refers to the ability to have clarity in the process of selecting–discriminating among various stimuli, facets, and sub-systems of conscious experience. It also means the ability to perceive and respond to differences and various changes in the inner and outer environment. Discrimination is a latent feature of conscious awareness, usually running in the background and appearing in cognition, body energy, emotions, or perception only when necessary; it seems to be involved in most of the facets and systems of conscious experience. This is a pattern of experiencing that seems to have strong conceptual links with concepts such as viveka (Sanskrit concept meaning “discernment or discrimination”) and sati sampajañña (a concept from Buddhism, usually translated as “clear comprehension”).

#### Quality of Experience (41 Items)

This explores the quality of subjective conscious experience, modulated through present-moment attention and awareness, and a subjective sense of well-being. It is reflected in concepts such as comfort with discomfort, equanimity, freshness, connecting with the present moment, compassion for self, kindness, peacefulness, inner fluidity, continuity of conscious awareness, amazement and mystery of life, and depth of interconnectedness with people and life.

#### Spirituality-Harmony (36 Items)

This scale refers to the ability to live in harmony with ourselves, other people and life forms, nature, and the environment. It explores living styles based on a spiritual perspective, including the deep connection with life (Gianan, 2010), respect and gratitude for being offered the experience of being alive, inner peace, and interest for deep and authentic connections with other people and life.

#### Global Self-Identity (101 Items)

Global self-identity includes traits, skills, and abilities related to identity, self-system, one’s image of life, self-awareness, the ability to see oneself as objectively as possible, flexibility in ego-related thinking (e.g., the ability to make and appreciate jokes about the way we are), self-compassion, self-kindness, and awareness of goals/direction in life. This scale tests for a selection of meta-skills related to post-autonomous ego development and psychological maturity (Graves, 1970; Cook-Greuter, 2013), such as awareness of ego as a construct, awareness of subpersonalities, being aware and connected to the feeling of life flowing through, non-reactivity to inner experiences, sense of wonder regarding everyday activities, serving as catalyst for other people, use of language awareness, overall flexibility and acceptance of various types of experiences, and good present-moment awareness.

#### Language Use (42 Items)

Maintaining awareness of language use is a meta-skill and refers to the ability to use language in an intentional and conscious way. Language is so deeply embedded and automatized through learning and practice, that many speakers of any given language are not aware of the reality construction imposed on them by their language (Cook-Greuter, 2013).

#### Physical Self (36 Items)

Physical self refers to the capacity for awareness of one’s body and of the actual elements of the environment (environmental awareness). This scale includes various traits, skills, and abilities such as interoceptive awareness, body posture, tone of voice, awareness of senses (e.g., smell, taste, touch), psychosomatic connections (how the body is influenced by emotions and thinking patterns), detecting automatic movements of the body (e.g., automatic eating behaviors), the connection with one’s physical surroundings, using touch to gain information about the outer world, and interconnectedness with the natural environment.

#### Energy Self (38 Items)

Energy self is a label for the awareness of the variety of energy exchanges that occur in our physical body (owing to various forces or phenomena; e.g., chemical, electromagnetism, body heat, food processing, changes in proprioception, etc.) and not to refer to the idea of a spiritual invisible force field that is not detectable by physics. The energy body is the result of life processes doing “work” to maintain the functioning of a human being, perceived in various ways, e.g., emotions, feelings, and flowing sensations. Energy self is about having a consistent awareness of energy flows and managing the dynamics of energy so that energy flows effectively throughout the body. Emotional intelligence is a key element in the development of good emotional awareness (Heim, 2003).

#### Cognition Self (65 Items)

Cognitive self refers to the capacity for awareness of one’s own ideas and thoughts, of the cognitive flow in general. The cognitive experience is related to thought, reflection, judgment, patterns of understanding, ways of meaning-making, accessing, and understanding information provided by body senses, and emotions and social interactions. It includes specific traits, skills, and abilities such as systems thinking, intuition, awareness of cognitive filters, metacognition (Darling-Hammond et al., 2014), self-reflection, detection of cognitive biases (e.g., jumping to conclusions, labeling, projection), accepting indecision, flexibility in thinking, critical thinking, present-moment awareness, awareness of the limits of words (construct awareness), attention regulation, an ability to act with intention (choice), decision-making, noticing rumination, mindfulness, acceptance of multiple perspectives, cognitive openness, creativity, the ability to have a panoramic view (overview) of a specific topic or situation, and the ability to manage the flow of thoughts.

#### Non-conceptual Self (31 Items)

Non-conceptual self is the effect of the activation of witnessing awareness mode, generating meta-awareness and a smooth present-moment awareness. The witnessing awareness mode generates a fresh look into the present moment (Brazdau, 2014), where there is only a present-centered experience even if the content may relate to memories of the past or some thinking about the future. The witnessing awareness mode is a part of a new mirroring/self-reflective system that appears to be active on a large scale in the human race, an evolutionary feature, slowly developing in humans and perhaps in other life forms (Brazdau, 2014). The witness is neither a conceptual structure, mediated by words, nor a superego that analyzes what is happening. It is simply a mirroring process, a feedback process, active in all life processes on Earth and translated into our psyche as the experience of being alive as love, and wide-awake (Andrews, 1996). Some researchers call it fundamental awareness, cosmic consciousness, pure consciousness (Forman, 1990) or non-symbolic awareness. Non-conceptual experiences are accessible across a wide range of developmental levels, they do not belong to a “higher” developmental level (Martin, 2010).

The non-conceptual self scale includes features such as attending to the present moment, mindfulness, non-reactivity to inner experiences, comfort with emotionally neutral experiences, feeling of oneness, connectedness with the environment, and accepting all emotions as they come and go.

#### Social-Relational Interconnectivity (86 Items)

Social-relational experience refers to the capacity for awareness of the relations and connections with the people around us and the communities of which we are a part. This scale includes traits, skills, and abilities related to parental relationships, close relationships, social interactions, perception of others’ communication styles, detecting social deception, cognitive empathy, social intuition, flexibility in social behaviors, outrospection, awareness of in-out group stereotypes, cognitive openness when discussing matters with others, detecting the hidden agendas of people we listen to or talk to, conversational skills, detecting social cues and depth of inter-connection.

#### Inner Growth (81 Items)

Inner growth refers to the capacity for awareness of the process of personal development, transformation, and growth, including learning and being aware of transformational patterns and stages. Within inner growth journeys, we must defeat psychological inertia and modify the inner defense mechanisms that maintain ego stability. The self-identity is a consequence of evolution; therefore, the process of changing is not gratified by nature with joy but with fear and frustration. Some people tend to see this inertia as a negative aspect, or negative energies that “attack them.” Some negative emotions are, in fact, emotions resulting from system inertia that naturally defends the ego. However, there are also positive emotions (e.g., enthusiasm), released as an evolutionary support to overcome this inertia (Brazdau, 2018).

The inner growth scale covers traits, skills, and abilities related to the evolution of personality, paradigm shifts, unlearning and learning (through pain or by open learning), openness, the language updating process, accepting criticism, abandoning old perspectives and embracing new ones, noticing resistance to change, learning after peak experiences, detecting the cognitive biases related to learning (e.g., confirmation bias), resilience, awareness of one’s level of development (e.g., using spiral dynamics theory) and an ability to sustain new patterns of thinking/feeling, while old patterns slowly lose their grip (awareness of the effects of neuroplasticity).

#### Multi-Modal Integration (43 Items)

Multi-modal integration refers to the ability to detect and be aware of the connections between various systems that compose our psyche, such as body, energy, emotions, thinking, and social connections. Integration awareness is supported by the development of a diffuse attentional style that keeps various flows of information or perceptions together, in a synesthetic-like manner (Monsanto, 2013). The scale includes topics such as identifying cognitive sources for various emotions, detecting the effects in the body of emotional or cognitive processes, awareness of the effects of food on the psyche, and ability to use words to describe sensations in the body or emotions. The most common integration pattern is psychosomatic integration, resulting from interactions between the mind, body, and emotions.

#### Habitual Patterns (21 Items)

Being conscious is the opposite of being on autopilot. Studies showed that humans are on autopilot more than half of the time (Killingsworth and Gilbert, 2010) and do not realize they have lost their free will and their freedom to choose how to react to what is happening to them. Inner automation plays a crucial role in our everyday lives; but it is necessary to access our automatic programs-to “re-write” them by adding “the free will subprogram,” then allowing them to become automatic again. For people at the beginning of their inner journey, this may look like a state of hyper-vigilance, or a permanent self-reflection that requires permanent attention and energy to deal with what is happening. However, eventually, it becomes natural and automatic. This scale includes the ability to notice the habitual-automatic patterns of the body, detection of verbal stereotypes, modulation of the automatic flows of thoughts, learning from repetitive events in life, and noticing the impulsive reactions to various stimuli or triggers.

#### Awakening Skills (Experimental Scale Composed of 61 Items, 30 in Section 1 and 31 in Section 2)

Awakening journeys are a continuum of openings and insights about the functioning of life and nature. Awakenings are subjective discoveries about the natural functioning of the various systems that are part of our make-up (Costeines, 2009). People worldwide seem to have the same types of “openings/awakenings,” that are subjective and associated with individuals’ personal/cultural frames of reference. Awakenings can be learned, cultivated, and invited and are habituated through practice (Brazdau, 2018).

### Descriptive Statistics (*n* = 2,266)

For the entire questionnaire with 237 items, reliability analysis revealed a very good internal consistency: Cronbach’s α = 0.989, McDonald’s ω = 0.990. The descriptive statistics for the 15 patterns of conscious experience are provided in Table 4.

**TABLE 4:** Patterns of conscious experience.

**Patterns of conscious experience**	**Cronbach’s alpha**	**No. of items**	**Sum (Mean)**	**Mean standard error**	**Standard deviation**	**Skewness**	**Kurtosis**
Perspective-taking	0.966	61	288	0.84	40	–0.600	0.932
Clarity of discrimination	0.982	107	497	1.51	72	–0.545	0.784
Quality of experience	0.959	41	173	0.70	33	–0.266	–0.171
Spirituality-harmony	0.951	36	161	0.60	29	–0.458	–0.032
Global self-identity	0.980	101	454	1.46	70	–0.429	0.511
Language use	0.950	42	199	0.56	27	–0.601	1.144
Physical self	0.949	36	162	0.56	27	–0.481	0.280
Energy self	0.956	38	159	0.63	30	–0.244	–0.027
Cognition self	0.971	65	303	0.90	43	–0.555	0.910
Non-conceptual self	0.949	31	128	0.55	26	–0.235	–0.194
Social-relational interconnectivity	0.974	86	400	1.17	56	–0.538	0.911
Inner growth	0.974	81	375	1.14	54	–0.595	0.850
Multi-modal integration	0.963	43	198	0.67	32	–0.548	0.467
Habitual patterns	0.924	21	98	0.31	15	–0.645	0.919
Awakening skills (Section 1)	0.928	30	131	0.47	22	–0.253	0.093

*Descriptive statistics (*n* = 2,266). Descriptive statistics generated from raw data; Skewness standard error = 0.051; Kurtosis standard error = 0.103. CQ, Consciousness Quotient.*

For the total score, the Kaiser–Meyer–Olkin measure of sampling adequacy (0.998) indicated that factor analysis might be useful. Bartlett’s test of sphericity results were also acceptable (approximately χ^2^ = 321,209.234; df = 27,966). The statistical descriptives for the CQ-i total score were as follows: mean = 1,077.22 (standard error = 3.217); 95% confidence interval for mean: lower bound = 1,070.91, upper bound = 1,083.53; 5% trimmed mean = 1,080.90; median = 1,083.00; variance = 23,453.407; *SD* = 153.145; minimum = 238, maximum = 1,422; range = 1,184; interquartile range = 210; kurtosis = 0.693 (standard error = 0.103).

CQ-i half-split reliability was tested using Cronbach’s alpha, Spearman-Brown coefficient, and Guttman split-half coefficient. The test items were split in two parts automatically by SPSS. Split-half reliability: Part 1, 119 items, *r* = 0.977; Part 2, 118 items, *r* = 0.982. Correlation between forms: *r* = 0.940. Spearman–Brown coefficient: equal length = 0.969, unequal length = 0.969. Guttman split-half coefficient = 0.969.

Scales data were tested for reliability to determine the possibility to create a modular assessment structure (independent testing for each scale). The result showed that each scale is reliable, with some scales having a larger variability than others. Skewness was calculated for each scale, to check if the scale has a quasi-normal distribution of scores. The kurtosis index was included to explore whether the distribution is too peaked (Hair et al., 2017). The most substantial variability in scores was observed for non-conceptual self, quality of experience, energy self, spirituality-harmony, physical self, and awakening skills. The higher variability of these scales may be owing to the variety of developmental methods available and used worldwide, many of them related to mindfulness, meditation, non-duality movements, or spiritual groups. Skewness showed that only a few scales were slightly skewed and were at the threshold for being considered quasi-normal (skewness > 0.5): habitual patterns, language use, perspective-taking, and inner growth.

The patterns of conscious experience are inter-related, containing overlapping items (an item may belong to more than one scale); thus, the scales are highly correlated. Scales’ intercorrelations are presented in Table 5.

**TABLE 5:** Patterns/scales intercorrelations (*n* = 2,266).

	**0**	**1**	**2**	**3**	**4**	**5**	**6**	**7**	**8**	**9**	**10**	**11**	**12**	**13**	**14**	**15**
0 Total CQ Score	1	0.955**	0.976**	0.923**	0.919**	0.983**	0.927**	0.871**	0.918**	0.966**	0.889**	0.972**	0.973**	0.936**	0.910**	0.943**
1 Perspective-Taking		1	0.920**	0.840**	0.870**	0.943**	0.947**	0.750**	0.826**	0.949**	0.798**	0.963**	0.962**	0.847**	0.858**	0.872**
2 Clarity of Discrimination			1	0.858**	0.855**	0.939**	0.912**	0.895**	0.860**	0.970**	0.813**	0.955**	0.939**	0.962**	0.924**	0.912**
3 Quality of Experience				1	0.935**	0.943**	0.784**	0.790**	0.962**	0.833**	0.977**	0.861**	0.881**	0.835**	0.781**	0.916**
4 Spirituality-Harmony					1	0.917**	0.791**	0.782**	0.880**	0.831**	0.917**	0.883**	0.895**	0.817**	0.760**	0.926**
5 Global Self-Identity						1	0.900**	0.812**	0.943**	0.938**	0.921**	0.946**	0.970**	0.907**	0.896**	0.919**
6 Language Use							1	0.743**	0.786**	0.944**	0.733**	0.957**	0.905**	0.829**	0.856**	0.819**
7 Physical Self								1	0.776**	0.808**	0.764**	0.796**	0.796**	0.920**	0.843**	0.870**
8 Energy Self									1	0.847**	0.949**	0.860**	0.871**	0.850**	0.793**	0.896**
9 Cognition Self										1	0.782**	0.971**	0.946**	0.903**	0.894**	0.872**
10 Non-Conceptual Self											1	0.815**	0.850**	0.807**	0.747**	0.894**
11 Social-Relational Interconnectivity												1	0.942**	0.882**	0.870**	0.897**
12 Inner Growth													1	0.901**	0.906**	0.897**
13 Multi-Modal Integration														1	0.931**	0.896**
14 Habitual Patterns															1	0.820**
15 Awakening Skills (section 1, 30 items)																1

** Correlation is significant at the 0.01 level (two-tailed).

### Score Classification

**FIGURE 2:**
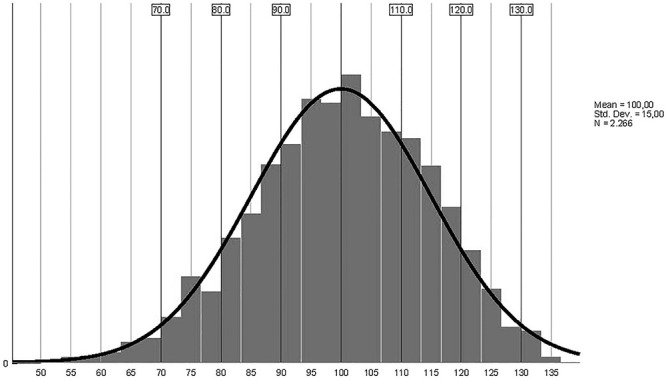
CQ-i Standardized scores – distribution curve (*n* = 2,266).

As the raw score distribution was quasi-normal, we decided to use standardized/transformed scores (transformed *z* scores, *M* = 100, *SD* = 15). The initial classification was split into five-point ranges for a better examination (Figure 2). The data for the first and second standard deviations were also used, and the final decision was to use six 10-point intervals, with the average range explored in two intervals: 90–99 and 100–109 (Table 6).

**TABLE 6:** Score classification table (*n* = 2,266).

**Score category**	**Consciousness Quotient score**	**% of individuals in normative sample**
Emerging (significantly below average)	<80	9%
Basic (moderately below average)	80–89	15%
Balanced (average range)	90–99	24%
Well-balanced (average range)	100–109	25%
Enhanced (moderately above average)	110–119	19%
Heightened (significantly above average)	≥120	8%

The extreme score intervals were combined for scores below 80 and above 120 (even if the data show there is a statistically relevant granularity) as we wanted to limit the inherent discrimination of people with low scores. CQ-i scores were classified into six intervals with inclusive labels, that were selected to reflect the evolution in the capacity for being conscious. We limited the number of score categories for practical purposes so that the test-takers would get a meaningful interpretation of their scores. The 6-level classification is the same for the global score and the scales scores. Future studies on larger samples are necessary for calibrating the cut-offs to more diverse populations.

## Discussion

### The Functional Role of the 15 Collective Patterns in Modulating Conscious Experience

Within the current model with 15 patterns of conscious experience, a possible perspective could be the following classification, based on EFA data and their functional purpose within conscious experience, as explored during the conceptual meta-research (Brazdau, 2018):

• Content providers: global self-identity, physical self, energy self, cognition self, and non-conceptual self;
• Selection and discrimination: perspective-taking, clarity of discrimination, and multi-modal integration;
• Evolutionary modulation: inner growth, awakening skills, and habitual patterns;
• Interconnectedness adaptation: social-relational interconnectivity and language use; and
• Harmonizing: quality of experience, and spirituality-harmony.

For the moment, this is just a theoretical hypothesis based on the inter-correlations between factors and the previous conceptual research on conscious experience. Additional future data from correlational studies are necessary to better describe the dynamics of this functional model with highly inter-related patterns.

### CQ-i and Psychological Maturity (Ego Development Stages)

The developmental model that relates conceptually to the CQ modeling is the Ego Development Theory by Cook-Greuter (2000) that includes some previous inquiries on maturity (Graves, 1970; Loevinger, 1976; Kegan, 1994; Martin, 2010; O’Fallon, 2015; Wilber, 2016) in an experience-based and testable model. The CQ-i includes items assessing many skills and capacities detected by Cook-Greuter’s research, transformed into behaviors and attitudes toward the experience itself.

The response scale, with six degrees, allowed us to observe the emergence of some psychological features, even in conventional stages. This comes from the observation that many post-conventional styles of being may be accessed even in conventional stages, as momentary experiences. The possible correlation between stages of maturity and conscious skills could be explored in further studies.

### Conscious Experience and Higher-Order Phenomena

The operationalization used to describe conscious experience involves witnessing awareness; having a degree of choice when thinking, feeling, sensing, and interacting with ourselves and all that is around us (i.e., not being on autopilot); and the capacity to feel awake and alive. Witnessing awareness may be considered a higher-order phenomenon because it includes meta-awareness, seen as awareness of awareness (Josipovic and Malach, 2006). Cognition also has this meta self-reflection feature, usually known as metacognition. Emotions also have it-we can have a primary emotion (e.g., sadness), and a meta-emotion (e.g., happiness) concurrently. The hierarchical systems structure is ubiquitous in nature; therefore, it may be possible that every psychological process has a meta-structure (O’Fallon, 2015; Beyer, 2018). The CQ-i includes items referring to metacognition, meta-awareness, and meta-emotions; subpersonalities and awareness of their existence; and being aware and noticing self-identity patterns. In the CQ modeling, these meta phenomena are just descriptors of conscious experience, among many others.

### CQ-i Usability for Educational and Research Studies

Although some exploratory studies revealed a possible predictive and incremental value of the CQ-i, related to various variables, e.g., executive functioning, academic achievements, yoga practices, pro-environmental behaviors, etc. (Brazdau and Mihai, 2011; Jones, 2012; Chauhan et al., 2013; Ahuja, 2014; Tuazon, 2014; Ahuja and Sharma, 2015; Poultney and Fordham, 2018) this was not our primary research purpose. Other studies using the CQ-i in various contexts provided additional data for questionnaire properties, and possible cross-cultural predictive validity (Raundeley and Kumari, 2016; Sharma et al., 2016; Sattar and Saaed, 2019). Considering that the theoretical model aligns with the collective patterns of being conscious, further research is necessary to establish the predictive and incremental value of the CQ-i. We are also interested in gathering more data about the dynamics of each of the 15 factors, as they could provide more specific correlations and predictions.

The CQ-i assessment platform is available online at www.consciousness-quotient.com. We invite students, researchers, trainers, and educators to explore the patterns of conscious experience by using the CQ-i in their researches, either by using the entire questionnaire (268 items) to explore the total score and the 15 scales or by customizing the assessment and using just one or more scales to examine specific patterns of conscious experience. The testing platform allows researchers to manage the participants, send invitations by email, monitor the online completion status, input data from the paper-pencil assessment, and export the dataset with the standardized scores. The CQ-i can also be used as a self-exploration tool by organizations interested in enhancing the conscious skills of their leaders and team members, as the questionnaire may be administered during training or personal development sessions, or as a part of professional or coaching programs.

### Conscious Experience as a Psychological Variable

In this research, our approach was focused on studying the conscious experience, not the philosophical concept of consciousness. From the psychological perspective, the conscious experience is just another variable that describes first-person experiences (Baars et al., 2003; Petitmengin, 2009). We did not analyze theories of consciousness or propose a new theory of consciousness; instead, we indexed the repertoire of experiences already classified as “conscious/explicit.” In our view, consciousness is a natural stage in the evolution of life on Earth, so, instead of proposing a “unified theory of consciousness,” which is beyond our capabilities, we followed the procedure described by the assessment standards: indexing first-person reports using a new questionnaire, adapting it to comply with the assessment standards, creating a list of experiences and situations, asking individuals to reply if their personal experience includes these situations and how often, then gathering a statistically significant sample, and detecting the patterns that already exist in human experience. Our purpose was to treat the conscious experience as a variable and find a way to use it in psychological assessment, to support the psychological research on consciousness by providing more sub-variables/scales. The multitude of frameworks used to collect facets and behaviors related to conscious experience may not be connected at a theoretical level. Still, they are all connected experientially, as they are all conceptual models referring to the first-person experience of being conscious.

### Limitations

We would like to emphasize that the CQ-i measurement is strictly focused on exploring to what degree people can tap into parts of their psyche that are already consciously available to them. Due to this limitation, at this stage of research, the results of this study need to be interpreted as mainly descriptive, meaning that the uncertainty, with regard to whether or not these results would replicate, has to be acknowledged. Future studies are necessary to see if the patterning model remains valid for larger and more diverse populations.

However, it is difficult to estimate if people who are not interested in personal growth would take the test. Until a more extended sample is gathered, we recommend the CQ-i be used exclusively for personal growth and as a tool for self-inquiry and self-exploration, either in personal work or in organizations.

Another limitation of this study is that, although the sample was large enough to be used for exploratory analysis, it was not large enough for doing a relevant confirmatory analysis, due to the amount of variance included in the variety of conscious experiences tested by the questionnaire. The sample size needed to perform a relevant Confirmatory Factor Analysis (CFA), based on the complex co-variances, has to contain at least 28,200 valid questionnaires. As we continue to gather data through the online assessment platform, CFA will be conducted when the required level of sample size will be reached.

## Conclusion

This study is an exploration of what it is considered to be conscious experiences by various models, using a self-report questionnaire, providing a conceptual framework for future in-depth studies on inner growth dynamics and psychological maturity. A set of 237 items covering all major aspects of subjective conscious experience was selected to detect the phenomenal patterns of subjective conscious experience. An EFA using a large sample (*N* = 2,360) revealed 37 axes, which were mapped into 15 scales, comprising the CQ construct. A sample with a quasi-normal distribution (*n* = 2,266) was employed for standardization and classification of scores similar to intelligence quotient/EQ coefficients (*M* = 100; *SD* = 15). The analysis of conscious experience models and data from the EFA showed that a multidimensional (modular) model with inter-related factors was needed. Periodic checks of the stability of the scales on which scores are reported will be conducted.

Following the studies on CQ-i usage in adolescents (Brazdau et al., 2014), we set the recommended usage to age ≥ 16 years. However, for paper–pencil assessment in classrooms on those aged < 20 or other educational contexts, we recommend administering the questionnaire in more sessions, using the “Pause assessment” options in the online assessment interface. There is no time limit for test-takers; the questionnaire is focused on qualitative aspects and requires a great deal of self-reflection and consideration of different perspectives, necessitating a good focus and a relaxed environment.

The CQ-i is ready for the next stages of research: exploring the collective patterns of conscious experiences on more diverse populations, detecting more nuances in patterns composition, and analyzing the inter-dynamics of conscious experience patterns.

## Data Availability Statement

The datasets presented in this study can be found in online repositories. The names of the repository/repositories and accession number(s) can be found below: Open Science Framework repository: https://osf.io/huwb4.

## Ethics Statement

The studies involving human participants were reviewed and approved by the Scientific Research Committee of the Faculty of Psychology, Ecological University of Bucharest, Romania. The CQ-i development research was included since 2005 as a research project in the research plan of the faculty, without any financial implications. Data collection and processing were strictly controlled, facilitated by the usage of the online assessment platform. Personal/socio-demographic data (email, country, age, education level) were used exclusively for statistical and psychometric analyses, and data access was strictly restricted to the researchers involved in the CQ-i development process. The patients/participants provided their written informed consent to participate in this study.

## Author Contributions

OB: conceptualization, methodology, validation, formal analysis, investigation, data curation, writing – original draft, and project administration. SA: conceptualization, methodology, validation, formal analysis, investigation, data curation, and writing – review and editing. C-DO: methodology and formal analysis. VJ: conceptualization, methodology, formal analysis, investigation, and writing – review and editing. SS: conceptualization, methodology, formal analysis, investigation, and writing – review and editing. CM: conceptualization and investigation. SA: conceptualization, investigation, and writing – review and editing. KF: conceptualization and resources. All authors contributed to the article and approved the submitted version.

## Conflict of Interest

The authors declare that the research was conducted in the absence of any commercial or financial relationships that could be construed as a potential conflict of interest.

## Publisher’s Note

All claims expressed in this article are solely those of the authors and do not necessarily represent those of their affiliated organizations, or those of the publisher, the editors and the reviewers. Any product that may be evaluated in this article, or claim that may be made by its manufacturer, is not guaranteed or endorsed by the publisher.

## Footnotes

1
www.jamovi.org

## Appendix

### Sample Items

**Sample Items (Section 1)**

(64) When discussing a topic, I notice the ambiguities in the language of the people I talk with.

(65) I find it easy to assess when someone I know behaves differently, because of their momentary emotional state.

(66) I have moments when I feel that all human beings belong to one big family, even though we do not know each other.

(67) I find the answers to questions without being able to fully tell how I arrived at those answers.

(68) When discussing a topic, I notice when the person I am talking with is overconfident (as if they were an expert), even if they do not know too much about that particular topic.

(69) I can make and appreciate a joke about the way I am.

(70) When talking to some people, I feel like I am able to connect with them profoundly beyond rational, emotional, or physical levels.

(71) I prefer to use my own words when speaking to someone instead of quoting others to support my views.

(72) I think about how I can contribute to the progress of humankind.

(73) I feel that my main goal in life is to just be.

(74) When I think of a situation from the past, I remember most of my thoughts and emotions at that moment and many of the details.

(75) I notice when my mind grabs an idea and starts using it as a theme for thinking, creating related streams of thought.

(76) When I fail at something important to me, I keep things in perspective (look at the big picture).

(77) If a topic I am thinking about is not helping me at the moment, I can abandon it easily, without fighting with it.

(78) I realize when I meet important people or when I am in important situations that can help me to improve or change myself for the better.

(79) When I meet my friends, I prefer to discuss about how we think and how we experience life, instead of just describing the events that have happened in our lives.

(80) I allow myself to doubt my perceptions and my beliefs.

(81) When talking to someone, I am aware of whether I use verbal stereotypes/clichés.

(82) I realize when my thinking is influenced by my emotional states (emotional oscillations).

(83) When I am in a bad mood, I easily identify its source.

(84) I know when my life partner is momentarily focused on priorities other than our relationship, even if he/she is not telling me.

(85) I realize when somebody is trying to be someone other than the person they usually are.

(86) When I have an issue with something, I am able to distinguish between solutions based on gut feelings and those based on rational thinking.

(87) I (intentionally) allow myself to experience both positive and negative emotions.

(88) When talking to others I stay connected and empathetic, even when we experience an unpleasant emotion.

(89) When I look at a landscape, I easily see all the details (the colors, the shapes, the small things, the empty space surrounding and defining it).

(90) I empathize with people’s experiences and feel their emotion, even if that experience is new to me.

(91) I am comfortable even if I do not quickly find an answer to my problems.

(92) When people talk to me, I listen to them with my full attention and I do not anticipate/think about what I will say next, while they are talking.

(93) I easily adapt my emotional responses to various social contexts.

(94) I notice when I become resistant to things that annoy me and do not accept them as they are.

**Sample Items (Section 2, Awakening Skills)**

(240) I let myself fully experience life through my body.

(241) I am able to intentionally change my attention styles (my lenses) to interact with life and various experiences.

(242) It is easy for me to maintain my full attention as a wide-awake witness, and, at the same time, complete everyday tasks with total focus and involvement.

(243) I experience moments of unconditional love.

(244) I can easily detect if a person seems to be locked in a collective cultural mirage or they have awoken from cultural and civilization “hypnosis.”

(245) The energy of life flows through me as a natural feeling of love directed toward me and everything around me.

(246) I am aware of my awareness.

(247) I can easily pay attention to my attentional styles and intentionally use various attention styles, as part of my everyday life.

(248) I am aware of how all things are interconnected in systems and flows.

(249) My everyday life has an intense quality of “freshness,” as if each moment is completely new.

(250) I have a deep connection with my body, as if we have a love affair.

(251) I enjoy interacting with life, by watching the consequences of my actions and then adjusting my actions based on life responses.

(252) I am aware of how the food I eat influences my body in the hours after I ingest it.

(253) I feel life energy flowing through me.
